# ATP driven clathrin dependent entry of carbon nanospheres prefer cells with glucose receptors

**DOI:** 10.1186/1477-3155-10-35

**Published:** 2012-08-02

**Authors:** Ruthrotha B Selvi, Snehajyoti Chatterjee, Dinesh Jagadeesan, Piyush Chaturbedy, Bangalore Srinivas Suma, Muthusamy Eswaramoorthy, Tapas K Kundu

**Affiliations:** 1 Transcription and Disease Laboratory, Molecular Biology and Genetics Unit, Jawaharlal Nehru Centre for Advanced Scientific Research, Jakkur P.O, Bangalore, 560 064, India; 2 Chemistry and Physics of Materials Unit, Jawaharlal Nehru Centre for Advanced Scientific Research, Jakkur P.O, Bangalore, 560 064, India; 3 Confocal Facility, Molecular Biology and Genetics Unit, Jawaharlal Nehru Centre for Advanced Scientific Research, Jakkur, Bangalore, 560 064, India

## Abstract

**Background:**

Intrinsically fluorescent glucose derived carbon nanospheres (CSP) efficiently enter mammalian cells and also cross the blood brain barrier (BBB). However, the mechanistic details of CSP entry inside mammalian cells and its specificity are not known.

**Results:**

In this report, the biochemical and cellular mechanism of CSP entry into the living cell have been investigated. By employing confocal imaging we show that CSP entry into the mammalian cells is an ATP-dependent clathrin mediated endocytosis process. Zeta potential studies suggest that it has a strong preference for cells which possess high levels of glucose transporters such as the glial cells, thereby enabling it to target individual organs/tissues such as the brain with increased specificity.

**Conclusion:**

The endocytosis of Glucose derived CSP into mammalian cells is an ATP dependent process mediated by clathrin coated pits. CSPs utilize the surface functional groups to target cells containing glucose transporters on its membrane thereby implicating a potential application for specific targeting of the brain or cancer cells.

## Background

Nanomaterials are being currently explored for various biomedical applications. In particular, carbon based nanomaterials such as single walled and multi-walled carbon nanotubes are increasingly being used as drug delivery vehicles. For imaging and efficient drug delivery, these nanomaterials are often tagged with some fluorescent agents and antibodies [1-3]. We had earlier reported amorphous carbon nanospheres [4] derived from glucose which are intrinsically fluorescent, nontoxic and have the ability to deliver the drug molecules inside the nucleus. Detailed *in vivo* studies showed that they were effectively removed from the animal system within a month and hence could be considered as potential carrier vehicle for therapeutic applications. However, to exploit the complete therapeutic potential of any carrier, the mechanism of its entry, preference of cell types and retention in the system needs to be thoroughly investigated.

In this study, we report the mechanism behind the cellular entry of CSPs and therefore it’s utility as a cell type specific targeting delivery agent. We have elucidated that CSP entry is predominantly a clathrin mediated and ATP dependent endocytic process. The rich functional surface groups and the charge on CSP gives them a unique ability to preferentially target cells with more glucose transporters such as the glial cells thus strengthening the possibility of CSP to be used as a potential drug delivery system targeted to the brain.

## Results and discussions

**Figure 1:**
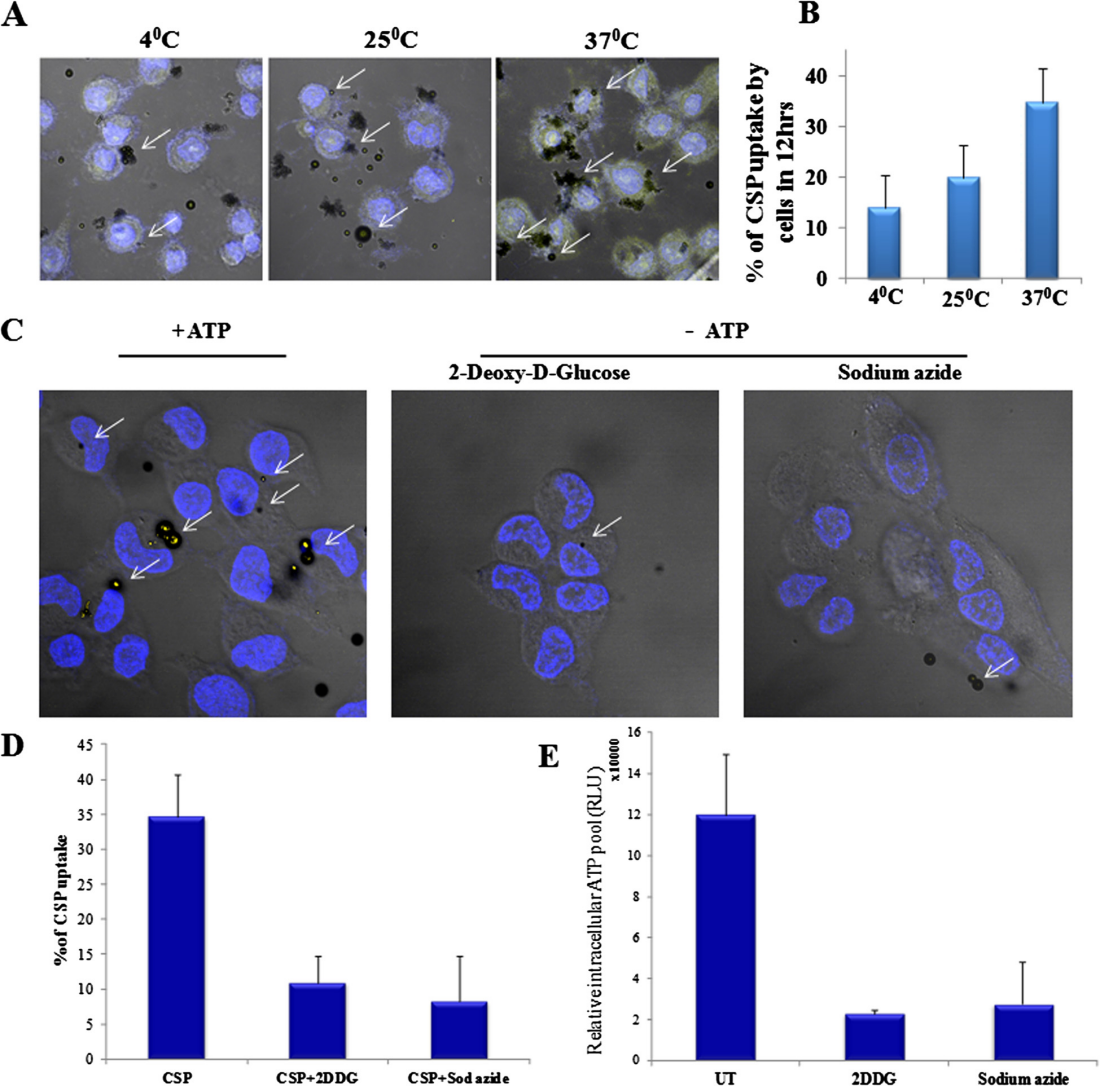
**The CSP uptake is an energy dependent process. A.** CSPs were incubated with HeLa cells at different temperatures (4°C, 25°C, 37°C) for 12 hours and were then fixed with 4% paraformaldehyde. The cells were then stained with Hoechst and were imaged using confocal microscope. **B.** Quantification for percentage of CSP uptake. **C.** ATP dependence of CSP entry was monitored by incubating CSP with HeLa cells pretreated with 2-deoxy-D-glucose and sodium azide. The panels are representative images of the cells in the presence or absence of ATP. **D.** Quantification for percentage of CSP uptake. **E.** Quantification of the total pool of intracellular ATP by using an ATP quantification kit (Invitrogen).

Carbon nanospheres ranging from 100–500 nm in size were synthesized [4] and tested for their ability to traverse the mammalian cell membrane. The CSP exhibits a time dependent entry with respect to different cellular regions. Within 3 hrs of incubation at 37°C, CSP could enter mammalian cells [4]. The large size and charged surface of CSP rules out the probability of entry into mammalian cells through diffusion. The most common method of particle uptake by cells is either a passive diffusion mechanism or an active process involving the energy obtained after hydrolysis of ATP. Since, the passive diffusion allows only liquids, gases or very small particles; the uptake of the large size CSPs is not possible. This led us to speculate that the uptake of CSP into cells is through endocytosis which is an active process. This was investigated by using the human cervical cancer cell line, HeLa. To determine, whether the CSP uptake is by receptor mediated endocytosis process, CSPs were incubated with the cells at different temperatures [5,6] : 4°C, 25°C and 37°C for 12 hours. The quantification of CSP present in the cells reveals that at 4°C (upon 12 hrs incubation) only 8-9% is localized inside the cell, whereas upon increasing temperature of incubation, the percentage entry increased substantially. At 37°C, it was found to be around 35-40% considering total number of cells to be 100% (Figure 1A and B). As low temperature blocks receptor mediated endocytosis [6], efficient CSP internalization observed at higher temperature could be predominantly mediated by endocytosis. As it is known that in mammalian cells the ATP synthesis and utilization is maximum near 37°C [7] the uptake of CSP by the HeLa cells could be ATP hydrolysis derived energy dependent. The requirement of ATP for the uptake of CSP was investigated by using a medium containing sodium azide or 2-deoxy-D-glucose (2DDG) which leads to depletion of the intracellular pool of ATP [8-11]. The depletion of ATP level was confirmed by quantifying the total intracellular ATP pool using a luciferase based assay system kit (Figure 1E) [12,13]. Pretreatment with sodium azide or 2DDG blocked the entry of CSP into the cells (Figure 1C and D) suggesting the involvement of ATP in the uptake of CSP. Since the low temperature inhibits formation of the active form of clathrin pits [14]; the clathrin mediated endocytosis could be operative for CSP uptake.

**Figure 2:**
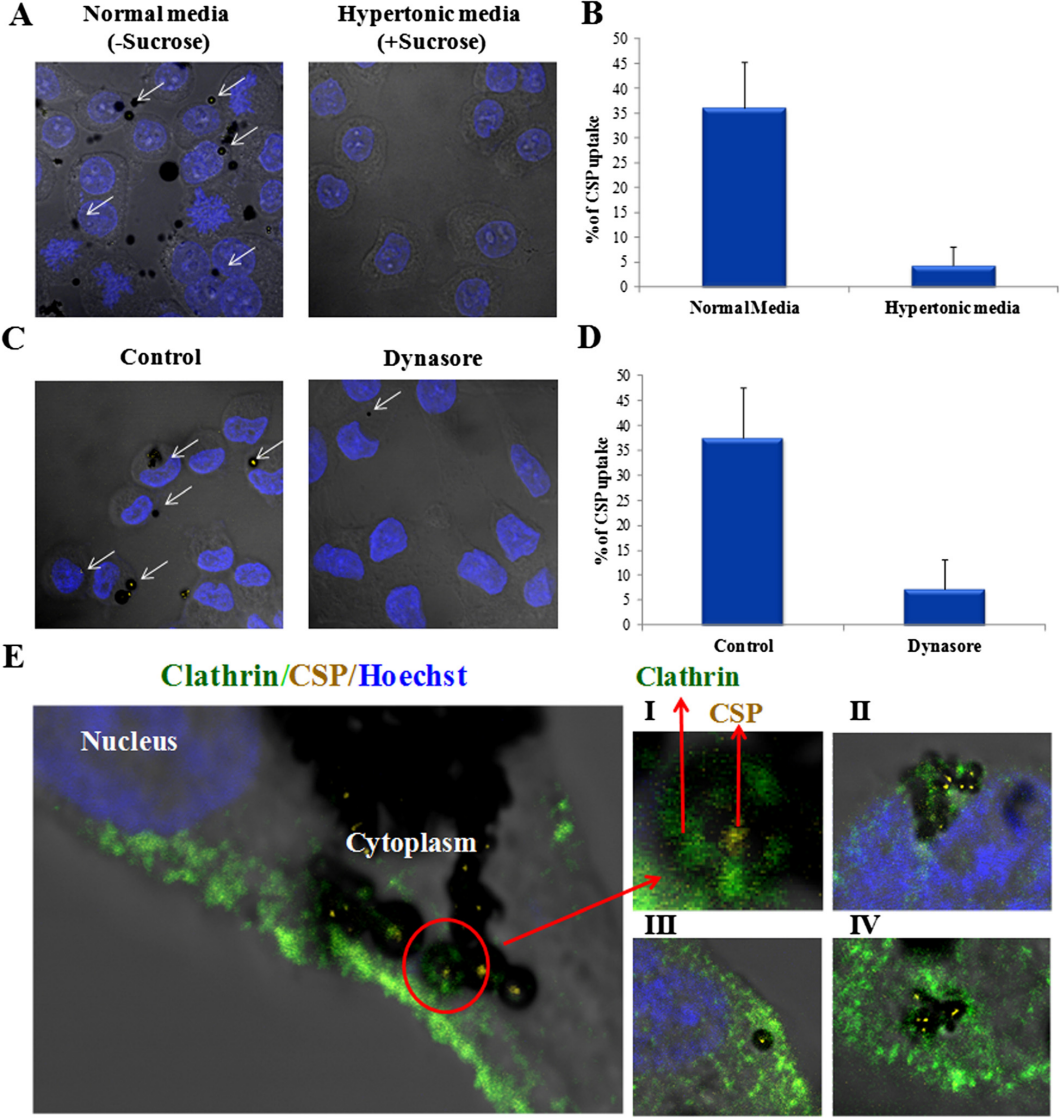
**The CSP uptake is mediated by clathrin dependent endocytosis. A.** HeLa cells were incubated with CSP in a hypertonic medium containing 0.45 M sucrose for 12 hrs and were then fixed with 4% paraformaldehyde. The cells were then stained with Hoechst and were imaged using confocal microscope**. B.** Quantification for percentage of CSP uptake. **C.** HeLa cells pretreated with or without 100 μM dynasore (an inhibitor of dynamin) for 30 mins and were then incubated with CSP for 12 hrs. **D.** Quantification for percentage of CSP uptake with or without dynasore treatment. **E.** Co localization of CSP with clathrin in HeLa cells, Panel I is the enlarged image of E, panel II-IV are independent replicates of E.

Endocytosis is the phenomenon by which cells internalize membrane proteins, lipids, extracellular ligands and soluble molecules. One of the earliest studied endocytotic pathways is the clathrin mediated process [15]. In addition, a variety of other clathrin-independent pathways have also been characterized including phagocytosis, macropinocytosis, caveolae- and raft-mediated uptake [16] and a poorly characterized form of constitutive clathrin-independent endocytosis. We performed a simple receptor blocking experiment by treating the cells with sucrose that created a hypertonic environment in the medium which prevents the formation of clathrin coated pits and thus further blocks receptor mediated endocytosis [17-19]. In agreement with the temperature dependent entry of the CSP into the cells, it was observed that hypertonic media [19] almost completely excluded the entry of CSP into mammalian cells incubated at 37°C (Figure 2 A and B). Clathrin mediated endocytosis pathways require dynamin which is required for the formation of coated vesicles [20] . To establish further the involvement of clathrin in the endocytosis of CSP, we treated the cells with a dynamin inhibitor, dynasore [21]. Pretreatment of dynasore for 30mins prevented CSP internalization into the HeLa cells (Figure 2 C and D). Furthermore, the colocalization of CSP and clathrin was also performed by fluorescence microscopy using anti-clathrin antibody to visualize the clathrin mediated endocytosis of CSP. Predominant localization of clathrin was observed in the regions enriched with CSP near the plasma membrane (Figure 2E). Collectively these results, suggests that CSP uptake by mammalian cells is an ATP dependent process that is primarily through clathrin mediated endocytic pathway.

We have previously observed that the CSPs by virtue of their surface functional groups [4] are capable of crossing the blood brain barrier (BBB), to elucidate the mechanistic details that facilitate the CSP entry across the BBB; we decided to test the CSP uptake on cell types present in the BBB as well as other tissue origins. For this purpose, we selected a mouse embryonic fibroblast NIH3T3 cell line, and cancerous cell lines of different tissue origins such as adenocarcinoma cell line of the neuronal origin SHSY-5Y, glioblastoma cell line U373MG and human cervical HeLa.

**Figure 3:**
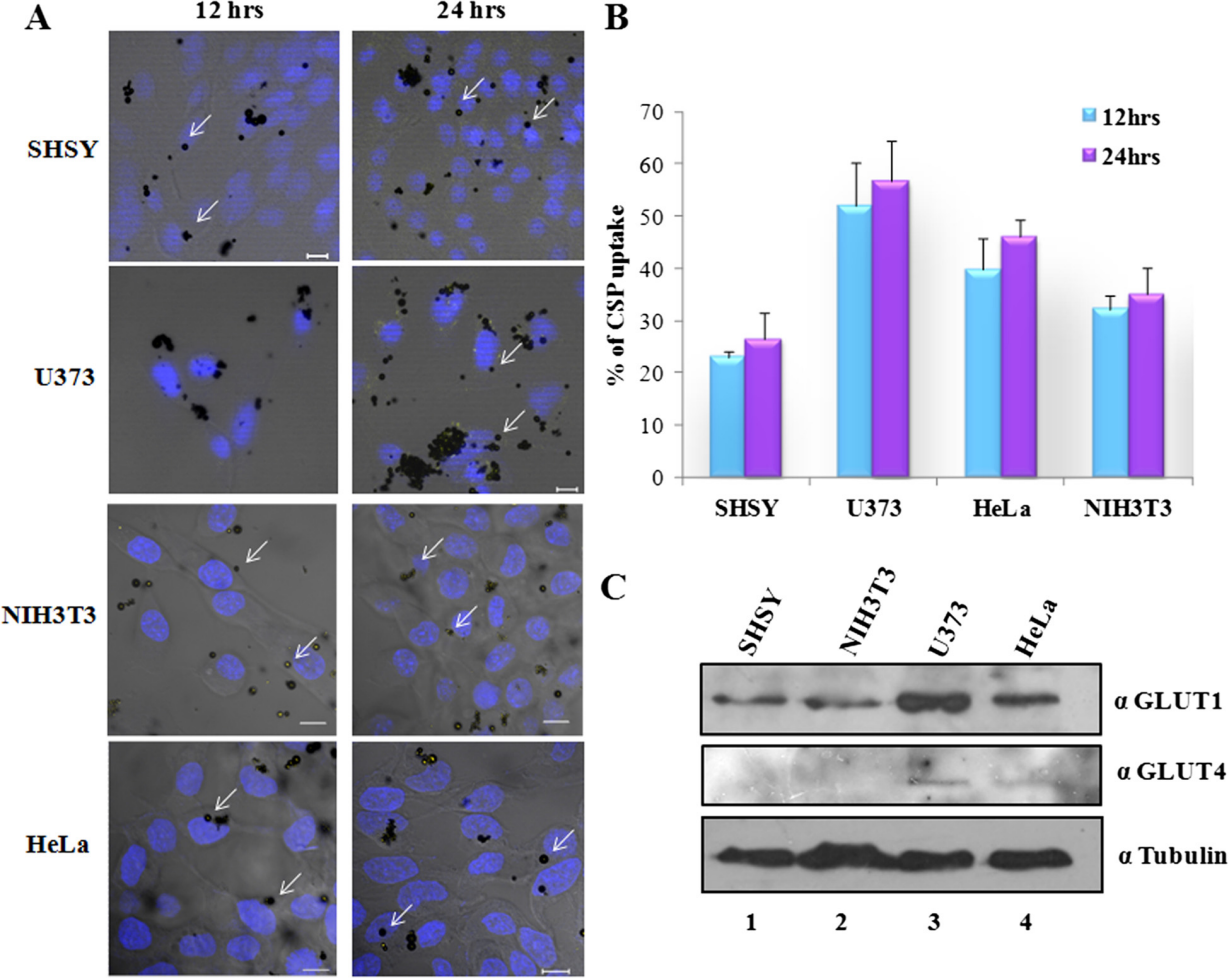
**CSP uptake across different cell types. A.** SHSY-5Y (adenocarcinoma cell line of the neuronal origin), U373 **(**glioblastoma cell line), NIH3T3 (mouse embryonic fibroblast) and HeLa (human cervical cancer) cells were incubated with 10 μg/ml of CSP. After either 12 hrs or 24 hrs, cells were washed with PBS and were fixed with 4% paraformaldehyde and were stained with Hoechst and were imaged using confocal microscope**.** CSP was visualized by exciting at 514 nm. Scale bar represents 10 μm. **B.** Quantification for percentage of CSP uptake by different cell lines at two different time points. **C.** Western blots for presence of GLUT1 and GLUT4 in different cell lines. Tubulin was used as a loading control.

As depicted in the figure, SHSY-5Y cells showed CSP uptake of 23%, NIH3T3 and HeLa cells showed CSP uptake ability of 32% and 39% respectively whereas U373MG cells showed 52% CSP uptake after 12 hrs time-point (Figure 3A). Even though there was no drastic difference in the cellular uptake of CSP between the two time points (12 and 24 h), uptake by U373 cell line was higher in comparison with NIH3T3 and SHSY-5Y cells, whereas uptake by HeLa cells was moderate (Figure 3B). There are several aspects that could contribute to these differences in the cell type specific uptake of CSP a possible reason could be density of glucose transporters on the cell surface. Immunoblotting analysis on U373 cells revealed that these cells express very high levels of glucose transporters GLUT1 and GLUT4 (Figure 3C). HeLa cells which also exhibit a moderate uptake of CSPs also expressed GLUT4 apart from GLUT1 which is present in all the tested cell types. GLUT1 is one of the most abundant glucose transporters present in the blood brain barrier and glial cells which facilitates transport of glucose molecules across the cell membrane [22]. The hydroxyl group of glucose molecule possesses high affinity towards GLUT1. Glucose molecules are involved in hydrogen bonding with the transmembrane alpha helices of GLUT1 [23]. As CSP contains free hydroxyl groups on its surface, it might interact with glucose transporters and facilitates the uptake of CSP.

**Figure 4:**
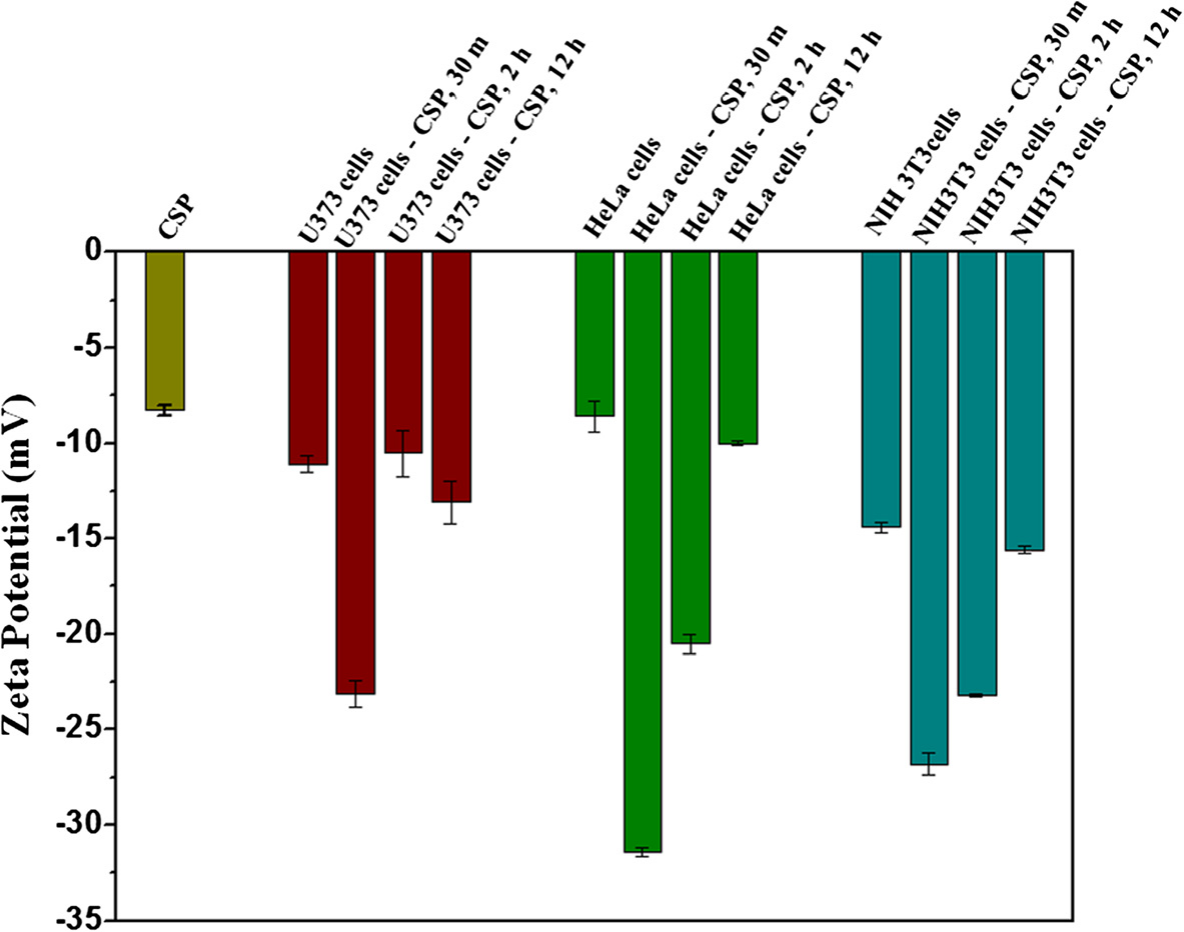
**Zeta potential of different cell type after treatment with CSP.** Zeta potential of different cell types viz. U373 cells, HeLa and NIH3T3 cells were measured in 40 mM HEPES buffer (pH 7.4) upon 30 minutes, 2 hours, and 12 hours of incubation with 10 μg/ml of CSP.

In order to support these observations we further carried out the cell surface charge measurements, which would be affected during the cellular uptake of CSPs. The cellular uptake of nanoparticles generally involves the binding of nanoparticles on the cell surface followed by internalization using a specific endocytosis pathway [24-26]. During this process, the cell zeta potential (a measure of its surface charge) would be changed. Cell membrane, in general, imparts a negative charge to the cell surface giving rise to a negative zeta potential for the cell. Binding of nanoparticles on the cell surface will change the zeta potential of the cell by, a) influencing the adsorption characteristics of the ions present around its surface and b) shifting the surface of hydrodynamic shear. When attached to the cell surface, negatively charged nanoparticles will make the cell zeta potential more negative, while positively charged nanoparticles will make the cell zeta potential less negative or positive. The internalization of nanoparticles by endocytosis leads to the cell zeta potential less negative. In order to study the interaction of the CSPs with different cell types, we carried out zeta potential measurements of the cell surface, after incubating them for different durations with CSPs. The zeta potential of CSPs in HEPES buffer (pH 7.4) is −8.0 ± 0.6 mV (Figure 4), which is less negative compared to the value obtained in pure water (−24 mV). The zeta potential value for U373 glial cells in HEPES buffer was found to be −11.1 ± 0.4 mV. Upon incubation of these cells with CSP for 30 minutes, the zeta potential became more negative, -23.1 ± 0.7 mV (Figure 4) which suggest that negatively charged CSPs are attached to the cell surface. Allowing the incubation time to 2 h, significantly brings the zeta potential of the cells close to their original value (−11.0 ± 1.0 mV), indicating the internalization of the CSPs. No notable variation in the zeta potential of these cells could be observed by increasing the incubation time from 2 hr to 12 hr (Figure 4, red bars). Thus the internalization process (of CSP) in U373cells completes within 2 hr of incubation. In contrast to the U373 cells which contains higher amounts of glucose receptor, a different trend was observed for NIH3T3 and HeLa cells (zeta potential in HEPES buffer), which contains lesser amounts of glucose receptors in comparison to U373 cells. Even though both the cell lines, HeLa and NIH3T3 took 12 hrs to completely internalize CSP, almost 50% internalization was observed in the 2 hrs timepoint for HeLa cells (Figure 4, blue bars) whereas CSP uptake was much lesser in NIH3T3 cells at 2 hrs timepoint (Figure 4, cyan bars). This difference in the changes of the cell zeta potential of different cell types for different duration of incubation could be explained by presence of the receptors on their cell membrane. Glial U373 cells are highly enriched with the glucose transporters GLUT1 on their surface while HeLa has moderate and NIH3T3 has the least levels. So, the affinity of CSP, which could mimic the glucose functionality on its surface, towards U373 cells will be very high as compared to the other cell lines. The zeta potential of U373 cells becomes highly negative by 30 minutes of incubation with CSP, suggesting it to be a receptor mediated adsorption, which results in preferred uptake of CSP by this cell line.

**Figure 5:**
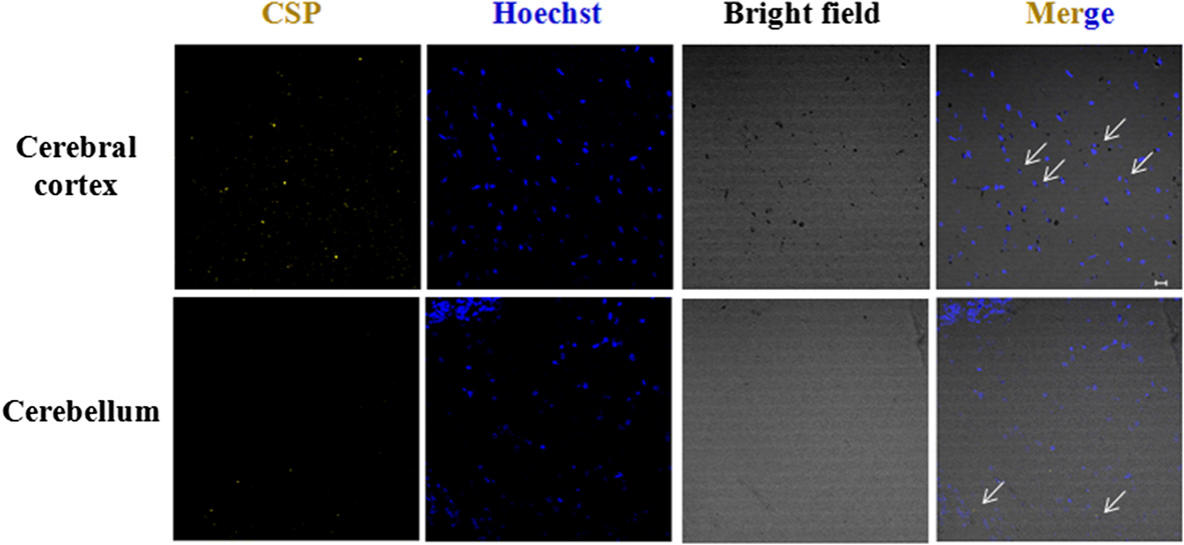
**Predominant localization of CSP in the cerebral cortex region of the brain. A.** CSP (10 mg per kg of body weight) was intraperitoneally injected in mice and after 3 days, they were sacrificed and their brains were isolated and immediately fixed in 4% paraformaldehyde. Sections of different brain regions were obtained and were processed for histochemical analysis. Hoechst was used to stain the nucleus and the cells were imaged using a confocal microscope. CSP was visualized by exciting at 514 nm.

Previously, it was reported that CSP could cross the blood brain barrier and enter the mice brain [4]. It was also observed in the liver and spleen, however, brain tissue showed maximum localization of CSP [4]. Since the mammalian brain is highly enriched with glia [27], and various glucose transporter proteins are also known to be localized on the brain capillary endothelial cell membranes that forms the BBB [28,29], the present observations of CSP preference to the glial cells and its preference towards glial cells also supports the previous report of increased brain localization, to be primarily mediated through the surface functionality of CSP. To further verify this preferential cellular targeting on specific brain cell types, the availability of CSP in different regions of the brain was investigated. CSP injected mice were sacrificed after 3 days and their brain was isolated, fixed with paraformaldehyde, sectioned and processed for confocal imaging analysis. Confocal microscopy of different brain parts (cerebral cortex and cerebellum) were performed for the presence of CSP by excitation at 514 nm. CSPs were predominantly localized in the cerebral cortex region with very minimal presence in cerebellum (Figure 5). We have observed an increased preference of CSP towards glial cells which could be correlated with higher population of glial cell lineages in the cerebral cortex of the brain than in the cerebellum [27,30,31].

These observations suggest that the glucose derived CSP by virtue of their surface charge possess unique ability to not just traverse the cell membrane but also exhibit a preference towards the cells with more number of glucose transporters on its surface. By virtue of its interaction with glucose transporters, it crosses the blood brain barrier and reaches the brain cells through the involvement of ATP dependent clathrin mediated endocytosis. ATP dependent clathrin mediated endocytosis plays fundamental role in neurotransmission and signal transduction [32]. Presence of higher levels of clathrin in brain in comparison to other tissue also partly answers the more specificity of CSP towards the brain [32,33]. Clathrin vesicles are also one of the major components of vesicle mediated endocytosis pathway in the synapses of various regions of brain involved in different phenomena [34-36]. Furthermore, predominant distribution of GLUT1 on the BBB that transfer glucose across the barrier is exploited by various neuroactive drugs to cross the BBB [37]. Thus GLUT1 in association with other glucose transporters might be related to the mechanism of CSP to cross the BBB. After reaching the brain through the glial cells, CSP may get transferred to other brain cells like neurons through association with other glucose transporters (presumably GLUT3).

## Conclusion

We elucidated the mechanism of CSP uptake by mammalian cells and found that it is predominantly clathrin dependent ATP driven process. CSP prefers cells expressing more number of glucose transporters thus providing increased specificity towards glial cells. CSP exploits its specificity towards glial cells for crossing the BBB, as glial cells containing GLUT1 are major components of the endothelial cells of the BBB. Once it reaches the brain it gets carried to various cell types including the neurons. Presently, when nanomaterials are being tested for their ability to deliver important drug molecules across different cells, tissues, a nanomaterial such as CSP which can preferentially target the brain cells, implies an important specific targeting mechanism which can be extensively exploited against several brain associated disease conditions such as the neurodegenerative states. Furthermore, the preference of CSP towards cell types with increased glucose transporters implicates its utility for targeting disease states wherein these levels are perturbed.

## Methods

### Cell lines

HeLa, SHSY-5Y, NIH3T3 and U373MG cells were grown in Dulbecco’s modified eagle’s medium with 10% FBS and antibiotics at 37°C in presence of 5% CO_2_ in a humidified chamber.

### Fluorescence studies

Cells were grown on poly L-lysine coated coverslips and were treated with 10 μg/ml of CSP. After 12 or 24 hrs of treatment, cells were washed in PBS and were fixed with 4% paraformaldehyde. Hoechst (Sigma) was used to stain the nucleus and the fluorescence was visualized using Carl Zeiss LSM 510 META laser scanning confocal microscope. CSP fluorescence was visualized at 560 nm by excitation at 514 nm.

### ATP depletion

HeLa cells were pretreated with 10 mM sodium azide and 30 mM 2-deoxy-D-glucose for two hours and were then incubated with CSP for 12 hrs. Following incubation cells were fixed with 4% paraformaldehyde. Hoechst (Sigma) was used to stain the nucleus and the fluorescence was visualized using Carl Zeiss LSM 510 META laser scanning confocal microscope.

### Determination of intracellular ATP levels

Intracellular levels of ATP in HeLa cells after treatments with 2DDG (Calbiochem) or sodium azide was determined by using an ATP determination kit from Molecular Probes, Invitrogen [12,13]. Briefly, cells were boiled in TE for 5mins before analysis and Luminescence was measured using Wallac 1409 Liquid scintillation counter.

### Clathrin blockage

HeLa cells were pretreated with media containing 0.45 M sucrose or 100 μM dynasore for 30mins and were then incubated with CSP for 12 hrs. Cells were then fixed and processed for confocal imaging.

### Mice brain immunofluorescence

BALB/c mice were injected intra-peritoneally with 10 mg per kg of body weight of CSP, 3 days post injection, they were sacrificed and their brains were immediately fixed in 4% paraformaldehyde. The brain tissues were dehydrated, paraffin embedded, and sectioned into 4 micron thick sections using a microtome (Leica). The tissue sections were stained with Hoechst and were visualized using Carl Zeiss LSM 510 META laser scanning confocal microscope. CSP fluorescence was visualized at 560 nm by excitation at 514 nm.

### Zeta potential measurements

The mammalian cells were incubated in the presence of CSP for different time points and were harvested by trypsinization. The cell pellets were resuspended in 700 μl of 40 mM HEPES buffer (pH 7.4). The zeta potential values of the cells and cells incubated with CSP for different time duration viz. 30 minutes, 2 hours, and 12 hours, were measured using Zetasizer Nano ZS (Malvern Instruments). All the measurements were carried out at 25°C using phase analysis light scattering mode. The Zeta (ξ) potential was calculated from the electrophoretic mobility based on the Smoluchowski equation, u = ϵξ/η, where u is the measured electrophoretic mobility, ξ is zeta potential value; ϵ is the dielectric constant η is the viscosity of the electrolytic solution.

## Animal ethical approval

All experiments using mice were followed according to the internationally recognized guidelines. The experiments were performed with the approval for ethical clearance from Institutional Animal Ethics Committee (IAEC), JNCASR, Bangalore, India (Reference number: IAEC/2011/TKK/002).

## Abbreviations

CSP: Carbon nanospheres; BBB: Blood brain barrier; ATP: Adenosine triphosphate; 2DDG: 2-Deoxy-D-Glucose; GLUT: Glucose transporter; HEPES: (4-(2-hydroxyethyl)-1-piperazineethanesulfonic acid; DMEM: Dulbecco’s modified eagle medium.

## Competing interests

The authors declare that they have no competing interests.

## Authors’ contributions

TKK was principal supervisor of this research in Jawaharlal Nehru Centre for Advanced Scientific Research, Bangalore, India. RS, SC and DJ carried out the experiments. DJ synthesized the CSP. RS, SC and TKK wrote the manuscript. ME from Jawaharlal Nehru Centre for Advanced Scientific Research, Bangalore, India contributed and supported in editing and completing this manuscript and gave us valuable guidance to improve this work. PC performed the zeta potential experiment and analyzed the results. BSS performed the confocal microscopy imaging. All authors read and approved the final manuscript.
